# Three-Dimensional Peri-Implant Tissue Changes in Immediately vs. Early Placed Tapered Implants Restored with Two Different Ceramic Materials—1 Year Results

**DOI:** 10.3390/ma16165636

**Published:** 2023-08-15

**Authors:** Malin Strasding, Yuwon Jeong, Laurent Marchand, Stefan P. Hicklin, Irena Sailer, Minji Sun, Hyeonjong Lee

**Affiliations:** 1 Division of Fixed Prosthodontics and Biomaterials, University Clinic of Dental Medicine, University of Geneva, Rue Michel-Servet 1, 1211 Geneva, Switzerlandirena.sailer@unige.ch (I.S.); 2 Department of Prosthodontics, College of Dentistry, Yonsei University, Seoul 03722, Republic of Korea; jyw5104@gmail.com (Y.J.); dr.mjsun@gmail.com (M.S.); 3 Clinic of Conservative and Preventive Dentistry, Division of Periodontology and Peri-Implant Diseases, Center of Dental Medicine, University of Zürich, 8032 Zürich, Switzerland; stefan.hicklin@zzm.uzh.ch; 4 Clinic of General, Special Care and Geriatric Dentistry, Center of Dental Medicine, University of Zürich, 8032 Zürich, Switzerland

**Keywords:** dental implants, bone-level tapered, immediate placement, early placement, peri-implant tissue, volumetric, digital dentistry, clinical study, multi-center RCT

## Abstract

Background: A prospective multi-center randomized controlled clinical trial was performed to digitally analyze tissue volume changes in immediately and early placed implants with simultaneous bone augmentation restored with two different all-ceramic materials. Methods: A total of 60 patients received 60 bone-level tapered implants (BLT, Straumann AG) immediately (n = 30) or early placed, 8–10 weeks after tooth extraction, (n = 30). Implants were restored with all-ceramic single crowns fabricated out of zirconia (Lava Plus, 3M), or lithium disilicate (E.max CAD, Ivoclar Vivadent AG) bonded to titanium base abutments (Variobase for Cerec, Straumann AG). Impressions were taken at baseline (BL), 6 and 12 months, and STL data were used to define an area of interest (AOI) to analyze peri-implant volume changes and midfacial recessions. Results: For immediate placement, a mean volume loss of −5.56 mm^3^ (±5.83 mm^3^) was found at 6 months, and of −6.62 mm^3^ (±6.56 mm^3^) at 12 months. For early placement, a mean volume loss of −1.99 mm^3^ (±5.82 mm^3^) at 6 months, and of −3.7 mm^3^ (±5.62 mm^3^) at 12 months was found. The differences in volume loss at 12 months between the two implant placement protocols were significant (*p* = 0.005). In both groups, mean midfacial recessions of 0.48 mm (±0.52) occurred. Conclusions: A more pronounced peri-implant volume loss can be expected 12 months after immediate implant placement compared with early placement.

## 1. Introduction

Over many decades, dental implants have proven to be a successful and predictable treatment option for replacing missing teeth [1,2]. High long-term clinical survival and success rates have led to dental implants often being the treatment of choice for single and multiple tooth restorations [3,4,5]. Furthermore, evolved implant geometry, surface topographies, and electrochemical coatings have improved primary stability and rapid osseointegration [6,7,8,9]. As a result, newer treatment options, such as immediate implant placement protocols, have become available [10,11,12]. The currently available literature indicates similar treatment outcomes for immediately placed implants compared with early or delayed placed implants [11,13,14,15,16]. At the same time, however, different bone healing patterns may influence peri-implant tissue volume changes. Therefore, bone augmentation procedures are used to preserve or increase hard tissue volume, and thus further stabilizing the peri-implant soft tissue [16].

When evaluating the esthetic outcome of dental implant treatments in the esthetic zone, the peri-implant soft tissue has, among many other factors, been described as one key factor for esthetic success. Several well-established, two-dimensional methods to investigate esthetic outcomes and mucosal recessions around dental implants have been described in the literature [17,18,19,20,21]. The midfacial mucosal recession was reported to depend on several factors, such as implant position, implant type and diameter, gingival biotype, and surgical technique [22]. Newer and more complex three-dimensional protocols have allowed for a more comprehensive analysis of peri-implant soft tissue volume [23,24]. Additionally, 3D radiographical assessments of peri-implant bone augmentation procedures and their volume changes are available [25,26,27,28,29,30,31]. Nevertheless, most currently available 3D analysis methods only present an overall peri-implant volume gain or loss, whereas a more detailed analysis method would be desirable.

Other factors potentially influencing the quantity and quality of the peri-implant tissues are the type and material of implant-borne restorations [32,33]. The configuration and surface topography of the supra-structure and the presence or absence of a submucosal cement gap may influence the peri-implant tissues [34,35,36]. Clinical studies have shown that the smooth surface of polished zirconia implant abutments demonstrated excellent biological integration with the peri-implant soft tissue [37,38].

However, more data is needed regarding the three-dimensional peri-implant tissue volume changes depending on the implant placement protocol, tissue augmentation procedures, and restoration material. Therefore, one aim of this multi-center prospective randomized controlled clinical trial was to digitally analyze the tissue volume changes in immediately and early placed implants restored with two different all-ceramic restorative materials.

## 2. Materials and Methods

The present study was performed according to the Declaration of Helsinki on medical protocol and ethics, in compliance with ICH-GCP guidelines and ISO 14155, and the CONSORT guidelines for randomized trials. The study was registered in the US clinical trials register (NCT05079542) on 15 October 2021. The protocol was approved by the respective local ethical committees (CCER 15-117).

For this multi-center randomized controlled clinical trial (RCT), patients were recruited at the University of Geneva, Switzerland (Division of Fixed Prosthodontics and Biomaterials) and in four associated private practices from 2015 to 2021. Sixty patients received 60 bone-level tapered (BLT) implants (Straumann AG, Switzerland) either immediately placed (group 1; n = 30) or by early placement 8–10 weeks after tooth extraction (group 2; n = 30). Simultaneous bone augmentation procedures were performed using autologous bone chips, a bovine bone substitute (BioOss, Geistlich Pharma, Wolhusen, Switzerland), and a resorbable collagen membrane (BioGide, Geistlich Pharma, Wolhusen, Switzerland).

The implant-borne restorations were all-ceramic single crowns fabricated from two different materials: zirconia (Lava Plus, 3M Schweiz GmbH, Rüschlikon, Switzerland) and lithium disilicate (E.max CAD, Ivoclar Vivadent AG, Schaan, Liechtenstein) bonded to titanium base abutments (Variobase for Cerec, Institut Straumann AG, Basel, Switzerland). The detailed study protocol was published previously [39]. Alginate impressions of the peri-implant region were taken at the final crown insertion, which was this study’s baseline (BL), and at 6 and 12 months after BL. A laboratory technician prepared cast models and then scanned them with a laboratory desktop scanner (IScan D104, Imetric 4D Imaging Sàrl, Switzerland) to create STL files for the volumetric analysis.

Horizontal and vertical volume changes of the peri-implant region were analyzed between BL and the different follow-up time points. A specific area of interest (AOI) was defined and applied to all models for this analysis.

### 2.1. Definition of the Area of Interest (AOI)

**Figure 1:**
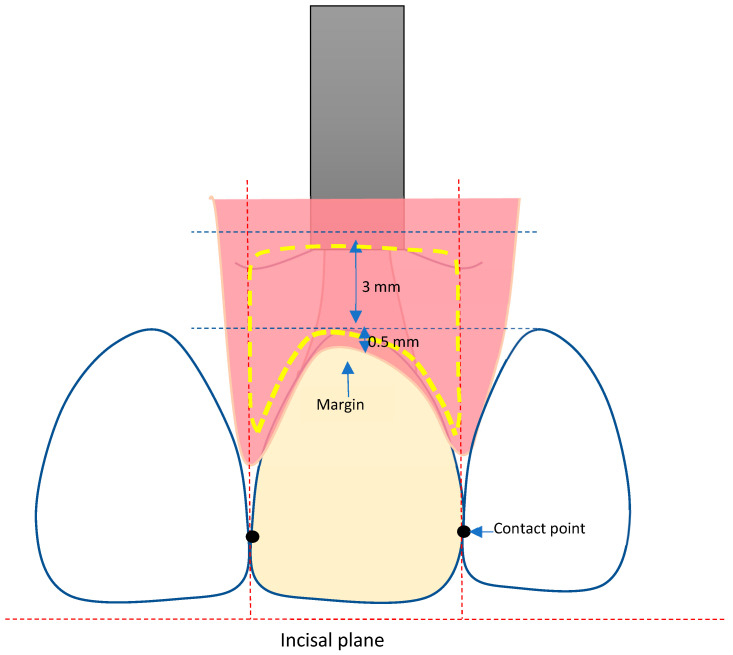
Definition of the AOI.

For the analysis of the peri-implant tissue volume changes, an AOI was defined and applied to all STL data in a standardized way (Figure 1).

The AOI was defined by tracing a line 0.5 mm below the mucosal margin, following the course of the gingival margin of the implant crown. For determining the mesial and distal borders of the AOI, vertical lines were drawn, going through the mesial and distal approximal contact points of the crown to the neighboring teeth and dividing the mesial and distal papilla into two halves each. The apical border of the AOI was defined by a horizontal line 3.5 mm below the zenith point of the crown.

**Figure 2:**
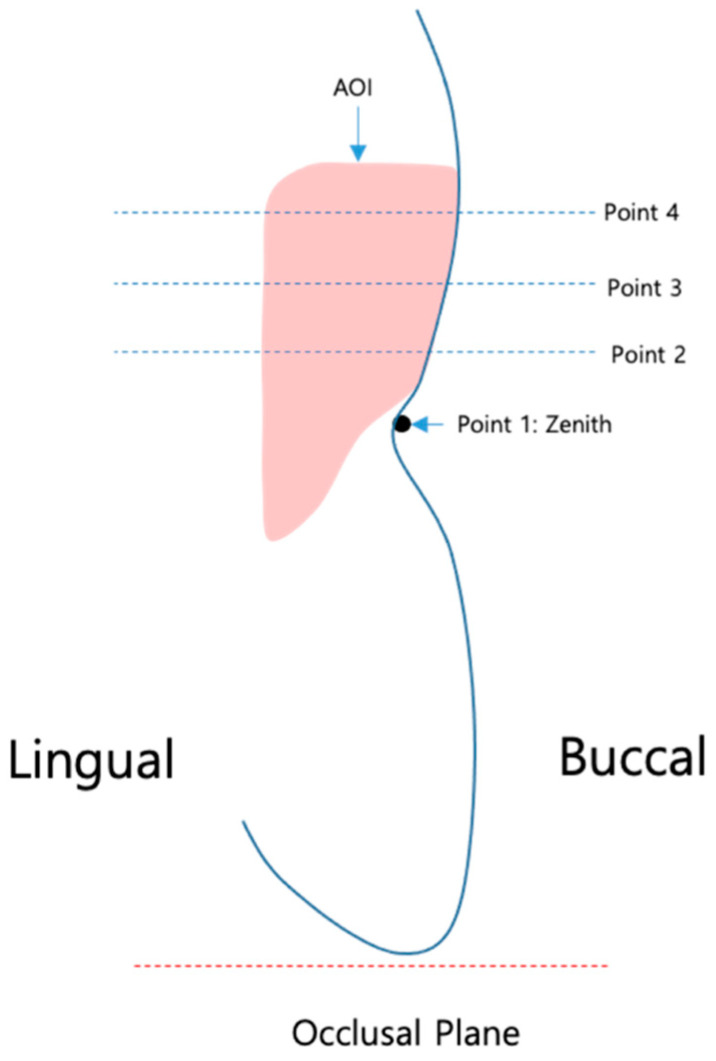
Defined position of points 1–4.

The midfacial mucosa height was defined as the distance between the incisal edge and point 1 = zenith. For the horizontal width measurements, three reference points were defined as reference points 2, 3, and 4, each 1 mm apart (Figure 2).

### 2.2. Definition of the Reference Points (Figure 2)

Point 1: reference point at zenith of the mucosal margin of the implant-borne crown.

Point 2: reference point 1 mm below zenith.

Point 3: reference point 2 mm below zenith.

Point 4: reference point 3 mm below zenith.

### 2.3. Volumetric Analysis

A previously developed method by Lee et al. was applied for this volumetric analysis of the peri-implant region using specialized software (GomInspect 2018, Gom, Braunschweig, Germany) [23]. This method allows detailed quantitative analysis of the volumetric changes at a specific site.

### 2.4. Analysis Procedure

**Figure 3:**
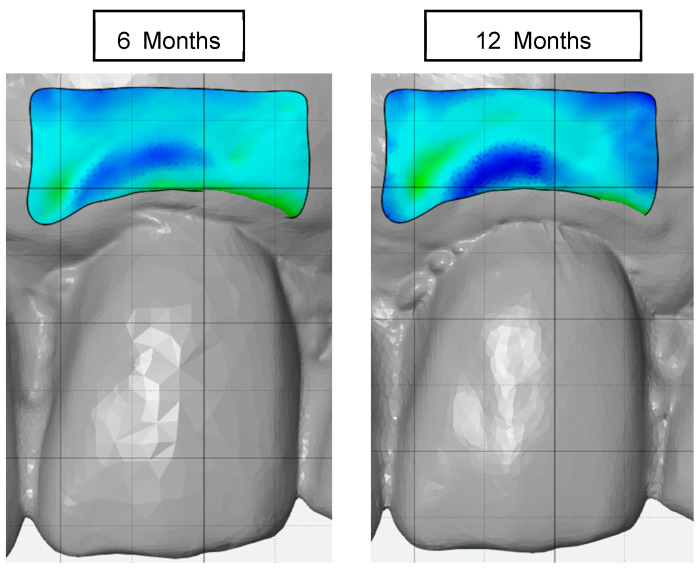
Selection of the AOI on 6-month and 12-month STL files.

**Figure 4:**
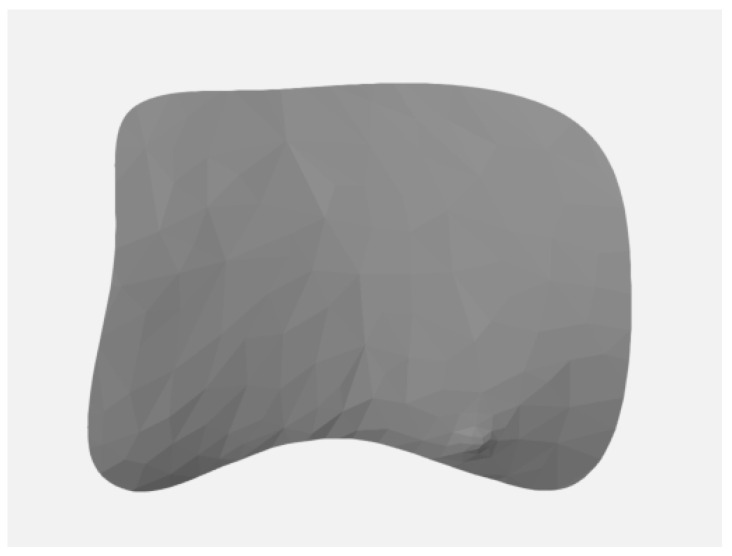
Separation of the AOI.

**Figure 5:**
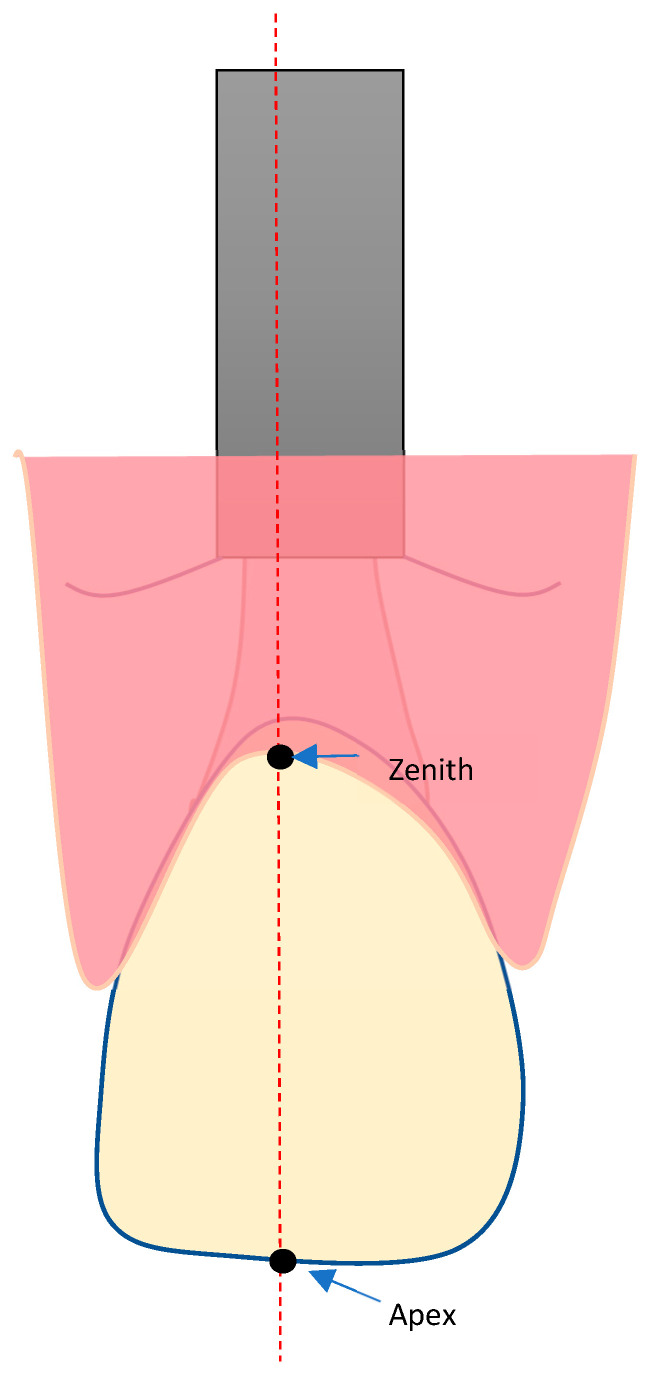
Selection of the reference point (= zenith) for the vertical marginal height analysis.

**Figure 6:**
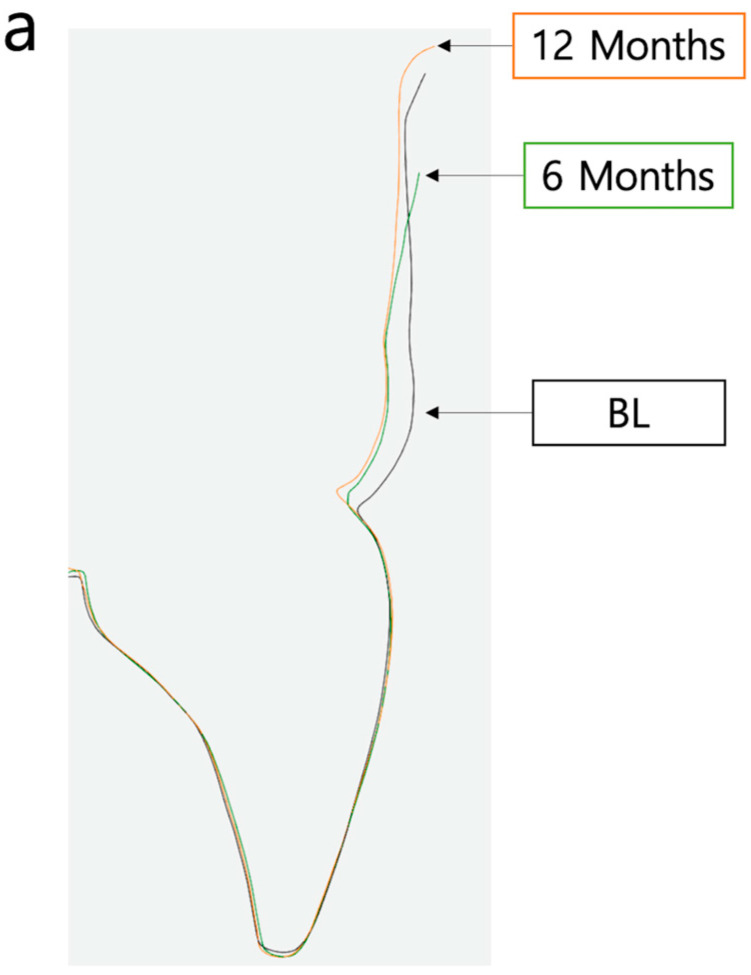

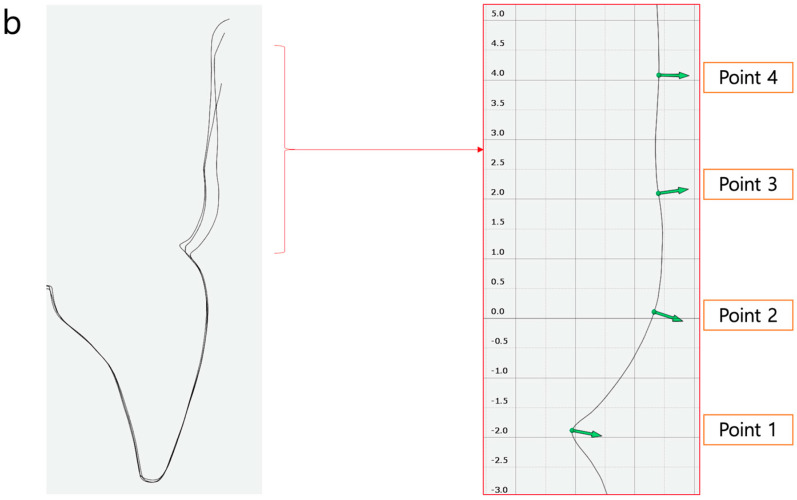
(**a**) Superposition of STL scan data of the different time points (BL, 6 months, 12 months) for visualization of tissue volume changes during follow-up visits. (**b**) Reference points 1–4 for analysis of zenith changes and horizontal thickness changes from BL to 6- and 12-month follow up.

**Figure 7:**
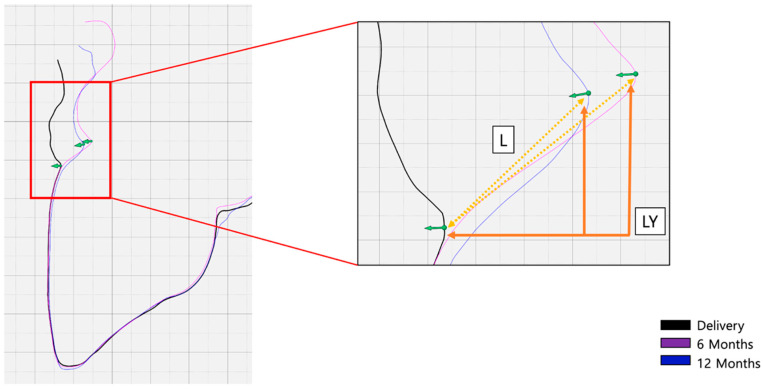
Analysis of zenith location changes (point 1) from BL to 6- and 12-month follow up.

1. Superimposition of STL data using best-fit algorithm focusing on the area of implant placement.
2. Alignment of the superimposed files with the x-, y-, and z-axes by each surface of the restoration of the implant.
3. Definition and marking of area of interest (AOI) in baseline STL and STL files at 6 and 12 months (Figure 3).
4. Separation of the AOI (Figure 4) from the original STL files.
5. Measurement of the AOI
6. Analysis by comparison of the AOI of each STL file: the separated AOI of the baseline visit was projected onto the AOI of the STL at 6-month and 12-month follow up to analyze any volume changes.
7. Vertical marginal height analysis using sectional analysis method. A vertical line was drawn from the zenith to the cusp/incisal edge of the tooth (Figure 5). Selection of reference points (points 1–4) on BL-STL was realized for analysis of horizontal changes in thickness at points 2, 3, and 4 and measurement of zenith changes (point 1) from BL to 6- and 12-month follow up (Figure 6 and Figure 7).
8. Analysis of volume changes from AOI of BL to AOI of 6 and 12 months, using the sectional analysis method described earlier [23].

### 2.5. Statistical Analysis

The measurements were analyzed using the statistical package SPSS Statistics 22 (IBM, Armonk, NY, USA). Normality was evaluated using the Shapiro–Wilk test, and a pairwise *t*-test was performed to compare the results of the two implant placement groups at 6 months and 12 months after baseline. The level of significance was at *p* < 0.05.

## 3. Results

Sixty patients, twenty-seven female (45%) and thirty-three male (55%), with a mean age of 56.6 ± 12.9 years, were enrolled in the study. All 60 subjects received one bone-level tapered (BLT) implant. Most frequently, premolars (48.4%), central incisors (21.6%), and lateral incisors (13.3%) of the maxilla were replaced, followed by maxillary canines (8.3%) and mandibular premolars (8.3%). For the present analysis, the baseline STL data of upper and lower jaw models of 56 patients were included.

One patient presented an early implant failure before implant loading; therefore, no STL model was available. Three other patients dropped out of the study during the follow-up visits as they could no longer be contacted, and their models were not included in the analysis. The models of 28 patients with 28 immediately placed implants and of 28 patients with 28 early placed implants were analyzed at BL. In the immediate placement group, 14 crowns were made of zirconia and 14 were made of lithium disilicate. In the early placement group, 15 were made of zirconia and 13 of lithium disilicate. Several scanned models could not be used for the analysis of the follow-up time points due to irregularities of the stone casts. Therefore, at the 6-month control, STL data of n = 51 patients and sites were available, whereas data of n = 47 patients and sites were available at the 12-month follow up.

### 3.1. Volume Changes

**Figure 8:**
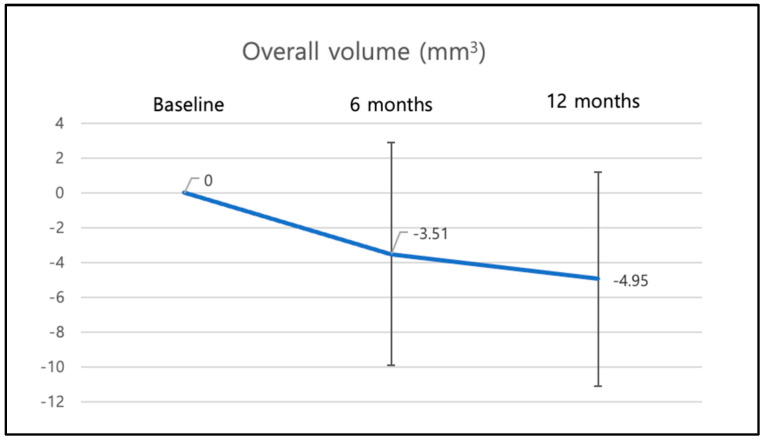
Overall volume changes (immediate and early implant placement) from baseline to 12 months.

The overall volume changes of the AOI from BL to 6- and 12-month follow up were analyzed. The mean overall AOI volume change from baseline to 6-month follow up was −3.51 mm^3^ (±6.39 mm^3^) and −4.95 mm^3^ (±6.16 mm^3^) to 12-month follow up (Figure 8).

**Figure 9:**
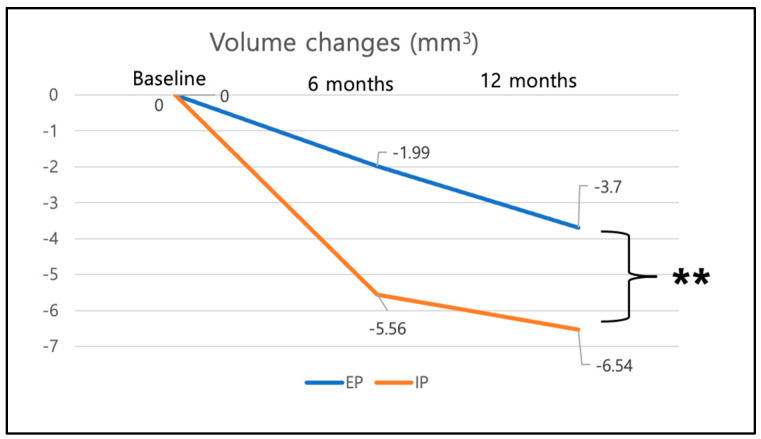
Volume changes between groups immediate vs. early implant placement at 6 months and 12 months. Significant difference at 12 months (*p* = 0.005) between the two groups. Statistical significance is marked as follows: ** = *p* < 0.01.

Furthermore, the AOI volume change from baseline to 6-month and 12-month follow up were analyzed between the groups immediate placement versus early placement (Figure 9). In the immediate placement group, a volume loss of −5.56 mm^3^ (±5.83 mm^3^) was found at 6 months and a mean volume loss of −6.62 mm^3^ (±6.56 mm^3^) at 12 months. For the early placement group, a mean volume loss of −1.99 mm^3^ (±5.82 mm^3^) at 6 months and of −3.7 mm^3^ (±5.62 mm^3^) at 12 months was found (Figure 9). Between baseline and 12 months, the immediate placement group (IP) showed significantly more overall volume loss in the AOI than the early placement group (EP) with *p* = 0.005.

No significant differences in volume changes were found between the two ceramic restorative materials (zirconia vs. lithium disilicate) with *p* = 0.972.

### 3.2. Overall Vertical Height Changes (Point 1)

**Figure 10:**
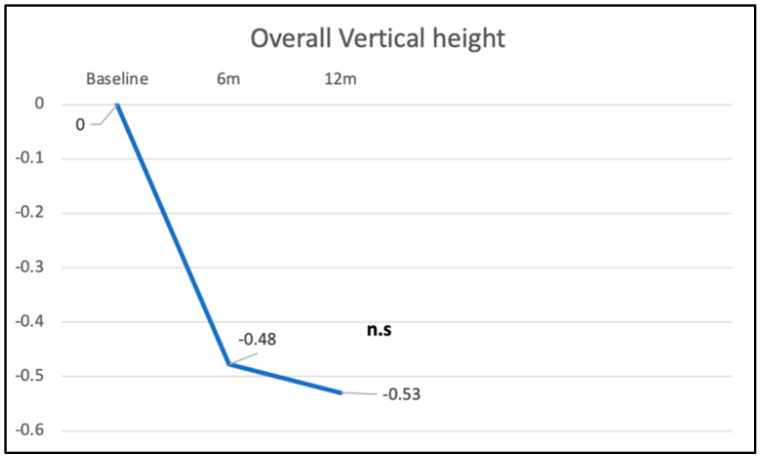
Overall vertical height changes (immediate and early implant placement) from baseline to 12 months. Differences were statistically not significant (n.s.).

The mean vertical height of the zenith point (point 1) showed a trend to negatively change slightly (*p* = 0.692) from baseline to 6 months of −0.48 mm (±0.52), which corresponds to a recession of the mucosal margin of 0.48 mm. From the 6-month to the 12-month follow up, a non-significant yet slightly negative trend in the zenith position from −0.48 mm to −0.53 mm (±0.49 mm) of −0.05 mm was found, which implies a slight tendency for further recession (Figure 10).

### 3.3. Vertical Height of Zenith Point in Immediate vs. Early Placement and Zirconia vs. Lithium Disilicate

**Figure 11:**
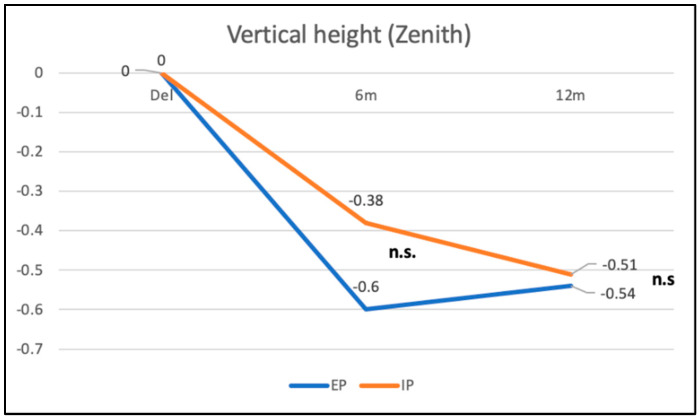
Changes in vertical height (reference point 1) for the immediate and early placement group from BL to 6-month and 12-month follow up. Differences between the groups were statistically not significant (n.s.).

In the IP group, the vertical height of the zenith point (point 1) was reduced from baseline to 6 months by −0.38 mm (±0.32 mm), corresponding to a recession of the mucosal margin of 0.38 mm. From the 6-month to the 12-month follow up, a slightly negative change in the zenith position from −0.38 mm to −0.51 mm (±0.43 mm) of −0.13 mm could be found, representing a tendency for continuing recession (Figure 11). The changes in vertical height were at no time points statistically significant.

**Figure 12:**
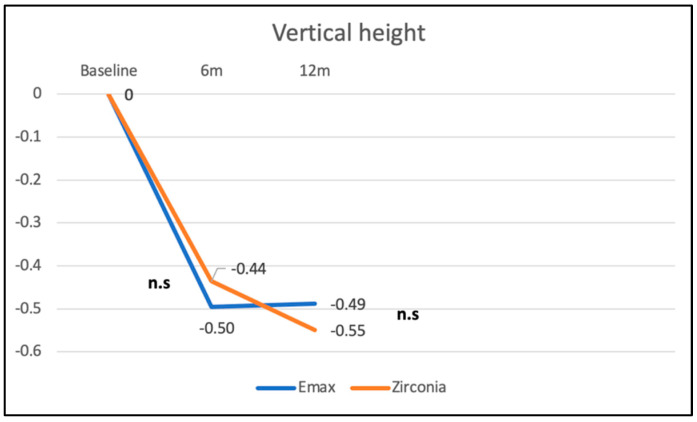
Changes in vertical height (reference point 1) for the lithium disilicate and the zirconia group from BL to 6-month and 12-month follow up. Differences between the groups were statistically not significant (n.s.).

In the EP group, the vertical height of the zenith point (point 1) was reduced from baseline to 6 months by −0.60 mm (±0.51 mm), corresponding to a recession of the mucosal margin of 0.60 mm. From the 6-month to the 12-month follow up, a slightly positive change in the zenith position from −0.60 mm to −0.54 mm (±0.36 mm) of +0.06 mm could be found, which implies a slight decrease in recession (Figure 11). However, these changes in the zenith point position for the different follow-up time points did not reach statistical significance. The differences between the two groups were not significant (*p* = 0.5). Furthermore, no significant differences in vertical height changes between the two ceramic restorative materials (*p* = 0.253) could be observed (Figure 12).

### 3.4. Changes in Horizontal Width

**Figure 13:**
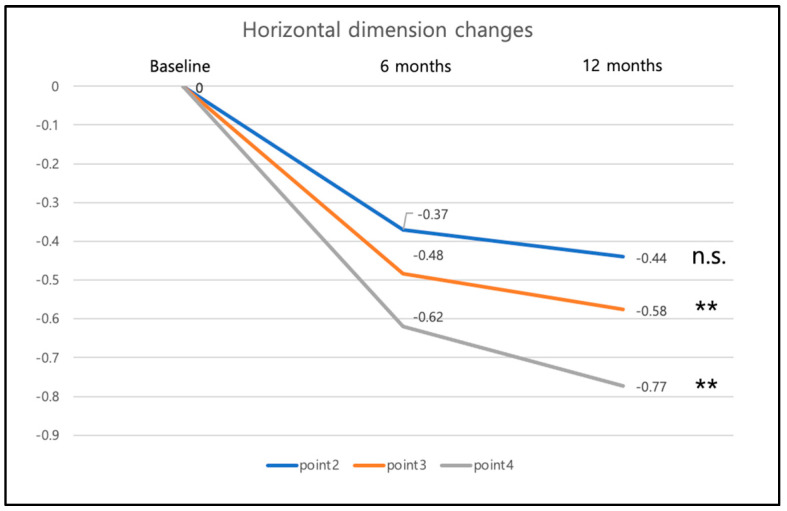
Overall horizontal dimension changes of the reference points 2, 3, and 4 at BL, 6-month and 12-month follow-up. Differences between the groups were statistically not significant (n.s.). Statistical significance is marked as follows: ** = *p* < 0.01.

The changes in horizontal width were measured at each reference point 2–4 (Figure 13): point 2 was found 0.37 mm (±0.46 mm) less buccally at 6 months and 0.44 (±0.49) mm less buccally at 12 months. This implies a decrease in horizontal width over time. Between the 6-month and the 12-month visit, the horizontal width decrease was not statistically significant.

Similar results were observed for points 3 and 4: point 3 was found 0.48 mm (±0.54 mm) less buccally at 6 months and 0.58 mm (±0.57 mm) less buccally at 12 months. Between the 6-month and the 12-month visit, the horizontal decrease was significant with *p* = 0.005. Point 4 was found 0.62 mm (±0.63 mm) less buccally at 6 months and 0.77 mm (±0.59 mm) less buccally at 12 months. The horizontal decrease was significant between the 6-month and the 12-month visit with *p* = 0.007.

**Figure 14:**
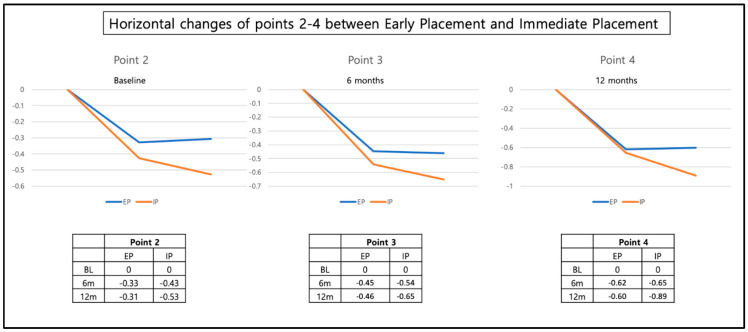
Horizontal dimension changes of the reference points 2, 3, and 4 at BL, 6-month and 12-month follow-up for immediate and early placement groups.

**Figure 15:**
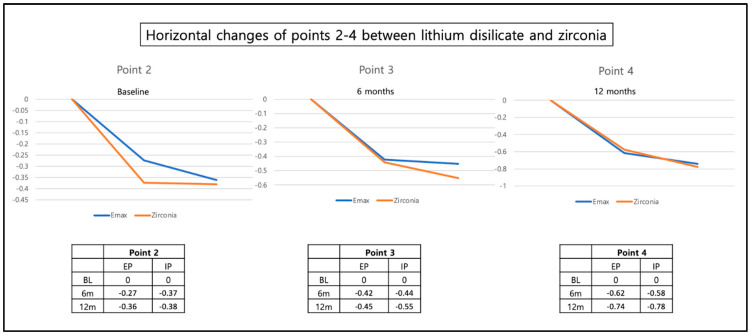
Horizontal dimension changes of the reference points 2, 3, and 4 at BL, 6-month and 12-month follow-up for Lithium disilicate and Zirconia restorations.

Neither the timepoint of the implant placement (immediate vs. early) nor the type of restorative material (zirconia vs. Lithium disilicate) had an influence (Figure 14 and Figure 15). The change in the horizontal position of reference points 2, 3, and 4 corresponds with a slight decrease in horizontal width in these regions.

## 4. Discussion

The present multi-center randomized controlled clinical trial demonstrated slight overall peri-implant volume loss following immediate and early implant insertion after one year of implant loading. A significant difference in peri-implant volume changes between the two placement groups was detected 12 months after loading. The early implant placement group showed less volume loss than the immediate implant placement group.

In the literature, the analysis of the stability of contour augmentation was often performed using radiographic CB-CT analysis after augmentation procedures [15,26,27,28]. Only a few studies analyzed the overall volume changes after implant insertion in three dimensions. Frequently, linear measurements were reported [40].

One study used a similar method as the present study [41]. The authors analyzed volume stability after implant insertion following an early implant placement protocol and soft tissue augmentation. Like the present study, alginate impressions of 15 patients were taken, and the resulting stone casts were scanned and transformed into STL data. The data were then superimposed, and volume changes were analyzed. The study group observed a volume decrease in the marginal area of the implant, whereas they found an increase in volume in the more apical part of their area of interest. Furthermore, they remarked on a high variability among their patient cohort. The present study’s data is in coherence with these findings of high inter-patient variability. In the mentioned study, the mean loss of facial prominence within the first year after crown insertion was −0.04 mm ± 0.31 mm. This distance corresponds to the change in volume over time and is the mean distance between all measured points in the bucco-oral direction. In the present study, the mean overall volume change for both groups was −5.1 mm^3^ after 12 months, corresponding to a mean bucco-oral distance of −0.3 mm at 12 months. No other studies were found that reported on three-dimensional volume changes (in mm^3^) similar to the present study after implant insertion and the bone augmentation procedure. Nevertheless, one study analyzing 3D radiographic data after immediate implant placement reported an almost complete loss of the buccal bone in one third of the 14 patients, which demonstrates a volume loss in the peri-implant area [27]. For this study, localized guided bone regeneration was performed during the immediate implant placement, likewise to the procedure performed in the present study.

The higher peri-implant volume loss in the immediate placement group compared with the early implant placement group can be explained by the expected bone remodeling and bone contour changes at post-extraction sites, especially within the first weeks and months after tooth extraction [40,42]. As volume changes were not analyzed before implant insertion, how much volume loss occurred in the early placement group after tooth extraction and before implant placement remains unknown. It can be assumed that a similar extent of volume changes occurred in both groups, but in the early placement group to an earlier time point, directly after tooth extraction and before implant placement.

Furthermore, midfacial recessions were observed in both groups after 6 and 12 months. In a study by Nimwegen et al., the presence of midfacial recessions after immediate implant placement and immediate insertion of the provisional restorations were compared between a study group receiving additional soft tissue augmentation (connective tissue graft) and a group not receiving soft tissue augmentation [43]. The results showed significantly higher midfacial recessions in the group without connective tissue grafting, with a mean difference of 0.68 mm between the study groups. In the control group with no connective tissue grafting, the mean midfacial recession amounted to 0.48 mm at 12 months in the latter study, which is equal to the present findings of a mean midfacial recession of 0.48 mm at the 12-month follow up [43]. Similar to the present study, another RCT comparing immediate and early implant placement with immediate insertion of provisional restorations in the anterior region found no statistically significant differences between the two groups [44]. Midfacial recessions amounted to 0.8 mm and 0.6 mm for the two groups, respectively, after two years of follow-up time.

One systematic review comparing midfacial recessions following immediate and early implant placement found notable deviations among the outcomes of the different included manuscripts [13]. The authors stated that immediate implant placement was associated with increased variability in the outcomes and a higher occurrence of midfacial recessions > 1 mm compared with the early implant placement protocol, where no recessions of >1 mm were reported. They concluded that immediate implant placement could increase the risk of midfacial mucosa recessions. The amount of detectable facial bone may influence the risk of midfacial mucosal recessions [13]. Furthermore, the bucco-oral position of the implant had an impact on the presence of midfacial recessions, with buccally placed implants significantly associated with recessions [13,45,46].

Overall, the presently reported extent of midfacial recessions after one year of 0.48 mm is in line with the expected average of midfacial recessions of 0.5 mm, for immediate and early implant placement, according to the comprehensive systematic review of Chen and co-workers [13].

In the present study, all implants were restored with a buccally micro-veneered all-ceramic crown made of lithium disilicate or zirconia and bonded to a titanium base abutment. This implies the submucosal contact of the peri-implant soft tissue with different restorative materials. After the 1-year observation period, no significant differences in peri-implant tissue volume changes were found when comparing the two ceramic materials. Several authors analyzed the short-term effects of various transmucosal restorative materials on peri-implant tissues, including the materials used in the present study, and found no significant differences regarding biological outcomes [47,48,49]. Similarly, several investigations demonstrated little to no long-term difference between metallic and ceramic implant abutments concerning the soft tissue inflammatory reaction [3,50,51,52]. Nevertheless, the evidence indicates that smoother material surfaces lead to less bacterial biofilm adhesion and, therefore, potentially improve peri-implant health [34]. However, more detailed long-term examinations of the peri-implant crevicular fluid analyzing inflammatory biomarkers such as interleukin or matrix-metalloproteinase of different restorative materials are necessary to provide clinical recommendations [53].

For the three-dimensional analysis performed in the present study, alginate impressions were made, and plaster models were cast, which were then scanned to obtain STL files. These digitized models were superimposed for digital volumetric analysis. An inherent shortcoming of this multi-step procedure is the risk of accumulated inaccuracies. A study using the same impression and data acquisition techniques to compare peri-implant volume changes after immediate implant placement with or without subsequent soft tissue grafting showed similar outcomes in inaccuracy as the present study [43]. For the present analysis, nine subjects had to be excluded from the analysis due to irregularities of the stone casts. Similarly, Nimwegen et al. excluded eight patients for the same reason [43]. This highlights the conventional impression techniques as a limitation of the chosen study design. A direct method of data acquisition using an intraoral scanner could reduce this risk and should, therefore, be preferred today.

The digital volumetric analysis method applied in the present study using the superimposition of STL data in specialized software has previously been described [23,54,55]. However, there is no consensus on a gold-standard technique for three-dimensional volume analysis in the currently available literature. This is shown by the heterogeneity of volumetric analysis methods used by other research groups. Other authors described the use of dental implant planning software not specialized in reporting volumetric outcomes [43,56].

Three-dimensional radiographic analysis stemming from cone-beam or micro-CT data is a widespread choice [57,58]. While excellent for the analysis of hard tissues such as teeth or bone, more evidence is needed to confirm the viability of this examination methodology for the precise inspection of the peri-implant soft tissues. In contrast, magnetic resonance imaging and subsequent volumetric analysis of soft tissues are standard in other fields of medicine, such as plastic and aesthetic surgery, but less common in dentistry [59].

## 5. Conclusions

This multi-center randomized controlled clinical trial showed that immediate and early placement protocols with simultaneous bone augmentation procedures lead to slight overall peri-implant tissue volume loss after one year. A significant difference in overall volume loss between the two time points of implant insertion was found. A slightly more pronounced peri-implant volume loss can be expected after immediate placement compared with early implant placement. Furthermore, slight midfacial mucosal recessions can be expected within the first year after implant placement.

Immediate placement protocols should therefore be avoided in esthetically demanding situations, such as anterior regions and high smile lines. It is essential to anticipate the post-extraction peri-implant volume and soft tissue changes and carefully choose between early and immediate placement protocols, depending on the patient’s clinical situation and esthetic demands.
